# Substrate cooperativity shapes competitive inhibitor responses in mycobacterial inosine 5′-monophosphate dehydrogenase

**DOI:** 10.1080/14756366.2025.2598479

**Published:** 2026-01-06

**Authors:** Zdeněk Knejzlík, Ondřej Bulvas, Matteo Dedola, Milan Štefek, Radim Nencka, Iva Pichová

**Affiliations:** Institute of Organic Chemistry and Biochemistry of the Czech Academy of Sciences, Prague, Czech Republic

**Keywords:** IMPDH, cooperativity, inhibitors, mycobacteria

## Abstract

Inosine 5′-monophosphate dehydrogenase (IMPDH) is a promising antimicrobial target due to its central role in guanine nucleotide biosynthesis. Accurate and reliable kinetic measurements are essential for evaluating inhibitors. However, the enzyme’s complex reaction mechanism and substrate cooperativity complicate analysis, leading to inconsistent reports on IMPDH reaction kinetics in key pathogenic mycobacteria. Here, we present an in-depth biochemical analysis of mycobacterial IMPDH, revealing pH-dependent cooperativity mediated by IMP-driven interactions between catalytic domains within the tetramer. This mechanism may result in paradoxical activation by IMP-competitive inhibitors under specific substrate conditions. We further show that such effects may influence apparent inhibition by the natural allosteric regulators GTP and ppGpp. Based on these findings, we outline practical recommendations for designing kinetic experiments that reflect physiologic conditions with the aim of more accurately evaluating IMPDH inhibitors for drug discovery.

## Introduction

The biosynthesis of purine nucleotides plays a critical role in cellular metabolism. Inosine monophosphate dehydrogenase (IMPDH) catalyses the NAD^+^-dependent conversion of inosine monophosphate (IMP) to xanthosine monophosphate (XMP), the first committed step in guanine nucleotide biosynthesis. IMPDH is highly conserved at both the sequence and structural levels across eukaryotes and prokaryotes. The enzyme comprises two distinct structural domains: a catalytic domain and a regulatory CBS domain (named after cystathionine-β-synthase). Due to its essential role, bacterial IMPDH is an attractive target for the development of novel inhibitors to combat infections caused by multidrug-resistant strains^1–3^. Targeting mycobacterial species with IMPDH inhibitors is a particularly promising strategy, supported by the identification of multiple inhibitor classes in recent years^4–7^. While the active site of IMPDH is highly conserved from bacteria to eukaryotes, making selective inhibition inherently challenging, successful attempts have been made to develop inhibitors that specifically target mycobacterial IMPDH^8^.

To accurately assess inhibitor effects, precise knowledge of enzyme kinetics is essential. The IMPDH catalytic mechanism, which has been extensively studied, involves two distinct chemical steps: dehydrogenation followed by hydrolysis. In the first step, the thiolate of the catalytic cysteine attacks the C2 position of IMP, forming a covalent E–XMP* intermediate while transferring a hydride to NAD^+^. In the second rate-limiting step, the reduced NADH leaves the active site, and the E-XMP* intermediate is hydrolysed to release XMP^9^^,^^10^. The complexity of bacterial IMPDH reaction kinetics is further increased by (i) substrate cooperativity towards IMP, (ii) a dynamic equilibrium between tetrameric and octameric states, and (iii) allosteric regulation by guanine and adenine nucleotide binding to the CBS domain, which modulates the conformation of the IMPDH octamer and, consequently, the enzyme’s activity^11^^,^^12^. To overcome some of these challenges, most inhibitor studies have utilised IMPDH variants lacking the CBS domain^2^^,^^7^^,^^8^^,^^13^.

Based on kinetic behaviour and oligomeric state, bacterial IMPDHs are classified into two distinct classes. Class I IMPDHs are characterised by a dominant octameric conformation and cooperative IMP kinetics, which are significantly modulated by ATP^14^. The best-characterised Class I enzyme comes from *Pseudomonas aeruginosa* (*Pae*IMPDH), which exhibits cooperative (Hill-type) IMP kinetics that shift to Michaelis–Menten behaviour in the presence of ATP, accompanied by a substantial increase in both IMP affinity and maximal reaction velocity^14^^,^^15^. In contrast, Class II IMPDHs exhibit Michaelis–Menten kinetics and undergo an ATP-dependent tetramer-to-octamer transition without a corresponding change in kinetic behavior^14^. The properties underlying this classification have been linked to the function of the CBS domains. A Class I *Pae*IMPDH mutant lacking the CBS domain exhibits kinetic properties comparable to those of the ATP-activated wild-type (WT) enzyme^15^. Moreover, experiments with chimeric IMPDHs, constructed by interchanging CBS domains between Class I and Class II enzymes, demonstrate that the CBS domain confers the characteristic molecular behaviour of each class^16^. However, the precise molecular mechanisms underpinning IMPDH cooperativity remain poorly understood.

Reports on the kinetic properties of mycobacterial IMPDHs have delivered conflicting findings regarding the presence of cooperative behaviour. While some studies describe Michaelis–Menten-type IMP kinetics^16^, others report cooperative behaviour. Rostirolla et al. reported pH-dependent Hill-type IMP kinetics for IMPDH from *Mycobacterium tuberculosis* (*Mtb*IMPDH)^17^. Consistent with this observation, we previously documented cooperative IMP kinetics for both *Mycobacterium smegmatis* IMPDH (*Msm*IMPDH) and *Mtb*IMPDH, accompanied by tetramer–octamer transitions in the absence of ATP-dependent activation^11^. These findings challenge the current classification of mycobacterial IMPDHs within the established Class I and Class II framework.

In this work, we further explore the mechanism driving mycobacterial IMPDH cooperativity, revealing a close relationship between reaction kinetics and the cooperation of the catalytic subunits. Through detailed kinetic analysis, we demonstrate that experimental conditions critically influence the evaluation of IMPDH inhibitors and that substrate cooperativity can lead to paradoxical activation by IMP-competitive compounds. Finally, we propose a set of recommendations to guide the design of experiments aimed at assessing the effects of IMPDH inhibitors.

## Materials and methods

### DNA constructs

Expression plasmids for full-length *Msm*IMPDH and *Mtb*IMPDH were constructed as described previously^11^. Briefly, coding sequences were amplified using Q5 High-Fidelity DNA Polymerase (New England Biolabs) and assembled with the In-Fusion HD Cloning Kit (Takara). The resulting plasmids were amplified in *E. coli* DH5α.

For *Msm*IMPDH (pRSF-HisTEV-MsmGuaB2), the full-length *guaB2* gene was amplified from *M. smegmatis* MC^2^ 155 genomic DNA using primer pair #1 + 2 and inserted into pRSF-HisTEV via the *SacI* site. The *Mtb*IMPDH construct (pET24d-HS_MtbGuaB2) was generated by amplifying *guaB2* from *M. tuberculosis* genomic DNA with primer pair #3 + 4 and cloning the product into PCR-linearized pET24d-HS (primers #5 + 6).

The expression plasmid for ΔCBS *Msm*IMPDH, in which residues of Ala111–Arg236 are replaced with a Gly–Gly linker, was constructed using the Q5 Site-Directed Mutagenesis Kit (New England Biolabs, USA) with primer pair #7 + 8 and plasmid pRSF-HisTEV-*Msm*GuaB2 as the template DNA.

All plasmids used in the study are listed in Table 1.

**Table 1.** List of oligonucleotides used in this study.

Primer number	Description	Sequence
#1	MsmGuaB2 WT for	5′-CAGGATCCGAATTCGGAAAACCTGTATTTTCAGGGCTCGATCGCTG AAGCAGCGTTCCCATCGCCG-3′
#2	MsmGuaB2 WT rev	5′-CCTGCAGGCGCGCCGTCAGCGGGTGTAGTAGTTGGGTG-3′
#3	MtbGuaB2 WT for	5′-GAACAGATTGGTGGGTCCCGTGGCA-3′
#4	MtbGuaB2 WT rev	5′-ATTCGGATCCGGTCTTTAGCGCGCGTAGTAGTTGGG-3′
#5	pET24d-HS for	5′-AGACCGGATCCGAATTCGAG-3′
#6	pET24d-HS rev	5′-CCCACCAATCTGTTGCGAT-3′
#7	MsmGuaB2 ΔCBS for	5′-GGAGGTCTGCTGGTGGGCGCGGCCG-3′
#8	MsmGuaB2 ΔCBS rev	5′-CTCGGACCGTTTGACCGTCTCGACCT-3′

### Protein expression

T7 promoter-driven protein expression was performed in *E. coli* BL21 LOBSTR cells^18^ (Kerafast, USA) using ZYM-505 expression medium^19^ supplemented with 50 μg/mL kanamycin. Cultures were inoculated at an initial OD_600_ of 0.05, grown at 37 °C to OD_600_ = 0.5, cooled to 18 °C, induced with 0.5 mM IPTG, and incubated for 20 h. Cells were harvested by centrifugation at 4000 × *g* for 20 min at 4 °C and stored at −80 °C until processing.

### Protein purification

*Mtb*IMPDH, *Msm*IMPDH, and ΔCBS *Msm*IMPDH were purified at 4 °C using ÄKTA FPLC systems (Cytiva). Frozen cell pellets were thawed and resuspended in lysis buffer (50 mM HEPES, pH 8.0, 500 mM KCl, 5% [w/v] glycerol, 5 mM β-mercaptoethanol, 10 mM imidazole, 1 mM PMSF, 0.1 mg/mL lysozyme) and stirred for 60 min at 4 °C. Cells were lysed by sonication, and lysates were clarified by centrifugation (38,000 × g, 30 min, 4 °C). The resulting supernatant was applied to a 5 mL HisTrap HP column equilibrated with buffer A (lysis buffer without PMSF and lysozyme). After washing with 10 CV of buffer A and 10 CV of buffer A + 60 mM imidazole, the bound protein was eluted with buffer A + 500 mM imidazole.

His-tags were cleaved by TEV protease (0.1 mg/mL, 1 h, room temperature), followed by overnight dialysis (12–14 kDa MWCO) against buffer B (10 mM K_2_HPO_4_, pH 8.0, 10 mM KCl, 5 mM β-mercaptoethanol, 10 mM imidazole). The dialysate was loaded onto a 5 mL HisTrap HP column to remove TEV, the uncleaved protein, and the free His-tag. To remove co-purifying nucleic acids, the flow-through was next loaded onto a 5 mL HiTrap Q HP column equilibrated with buffer B; the bound proteins were eluted with a gradient to buffer C (buffer B + 1 M KCl).

Target protein fractions were pooled and further purified by size exclusion chromatography on a HiLoad 26/600 Superdex 200 pg column in buffer D (200 mM K_2_HPO_4_, pH 8.0, 1 M KCl, 5 mM β-mercaptoethanol). Finally, the sample was exchanged into storage buffer (50 mM Tris-HCl, pH 8.0, 0.5 mM TCEP) using a HiPrep 26/10 desalting column, concentrated (30 kDa MWCO), aliquoted, flash-frozen in liquid nitrogen, and stored at −80 °C. Purity of the final *Mtb*IMPDH, *Msm*IMPDH, and ΔCBS *Msm*IMPDH samples was assessed by SDS–PAGE; identity was confirmed by mass spectrometry.

### Ribavirin 5′-monophosphate synthesis

Freshly distilled POCl_3_ (110 µL, 1.2 mmol, 3 eq.) was added to an ice-cold suspension of ribavirin (98 mg, 0.4 mmol, Carbosynth) in dry trimethyl phosphate (1.05 mL), and the mixture was stirred at 0 °C for 3 h. A solution of triethylammonium bicarbonate (TEAB, 2 M, 1.5 mL) was added; the mixture was then stirred at 0 °C for 15 min and at 25 °C for 2 h. Once volatiles were evaporated, the residue was co-evaporated several times with water. The product was purified using a combination of ion-exchange chromatography (POROS HQ, 0%–30% 1 M NH_4_HCO_3_ in water) and reversed-phase flash-column chromatography (C18, 0%–50% of ACN in 0.1 M aqueous TEAB). The sodium salt of the product (27 mg, 0.078 mmol) was obtained using Dowex 50 (200 mesh) on Na^+^ cycle. NMR characteristics corresponded with published data^20^. High-resolution mass spectrometry (HRMS) using electrospray ionisation in negative-ion mode (ESI−) gave [M − H]^−^ *m/z* 323.0398 (calculated) and 323.0395 (found) for C_8_H_12_N_4_O_8_P.

For ion-exchange chromatography (POROS 50 HQ Strong Anion Exchange Resin) and reversed-phase flash-column chromatography (C18 RediSep Rf columns), a Combiflash Rf from Teledyne ISCO was used.^1^H,^13^C, and ^31^P NMR spectra were recorded on a BrukerAvance III HD 400 instrument with a broadband PRODIGY cryoprobe and an ATM module (5 mm CPBBO BB-^1^H/^19^F/D Z-GRD). HRMS analysis was performed using an LTQ XL Orbitrap XL (Thermo Fisher Scientific) and ESI.

### Mass photometry analysis

Mass photometry measurements were performed at room temperature using a TwoMP automated mass photometer (Refeyn). Calibration was performed according to the manufacturer’s protocol using the droplet-dilution method in 20 µL droplets to measure 20 nM bovine serum albumin (BSA; 66 kDa monomer, 132 kDa dimer) and immunoglobulin G (IgG; 150 kDa monomer, 300 kDa dimer) to generate the calibration curve. *Msm*IMPDH WT and ΔCBS proteins were diluted to 20 nM in 50 mM Bis–Tris propane·HCl (pH 7.0–9.0), 200 mM KCl, and 0.5 mM TCEP buffer, and then incubated for 5 min prior to measurement. The effects of nucleotides on the oligomeric state were assessed at a pH value of 7.5 in the presence or absence of 5 mM ATP and 6 mM MgCl_2_. Scattering movies (60 s) were acquired and analysed using DiscoverMP software (Refeyn).

### Metabolomics

The whole-genome-sequenced *M. smegmatis* mc^2^155 laboratory strain was kindly provided by Dr. Libor Krásný (Institute of Microbiology, Czech Academy of Sciences, Czech Republic). Cells were cultured in Middlebrook 7H9 medium supplemented with 0.5% glycerol, 0.1% glucose, 0.5% bovine serum albumin, and 0.1% tyloxapol at 37 °C with shaking at 200 rpm.

For metabolite extraction, cultures were inoculated into 200 mL of medium in 1 L Erlenmeyer flasks at an initial OD_600_ = 0.001 and grown in three biological replicates. At defined growth stages, cells corresponding to a total OD_600_ of 2 were harvested by rapid filtration through 0.45 µm, 25 mm cellulose acetate filters and immediately quenched with ice-cold 1 M acetic acid, followed by flash-freezing in liquid nitrogen.

Nucleotide extraction and targeted mass spectrometry quantification were performed as described previously^21^, with minor modifications. Briefly, frozen filters containing bacterial cells were slowly thawed on ice and incubated for 30 min with vortexing every 5 min. The crude lysate was separated from the filter by centrifugation (4000 × g, 30 s) through a pinhole at the bottom of a 1.5 mL microtube. The lysate was flash-frozen in liquid nitrogen and lyophilised. The dried material was resuspended in 200 µL of ice-cold 50% acetonitrile and incubated on ice for 30 min. After centrifugation (22,000 × g, 20 min, 4 °C), the clarified supernatant containing the aqueous metabolite extract was collected and stored at −80 °C until analysis.

Metabolite separation was carried out on a cZIC-HILIC column (150 × 2.1 mm) at a flow rate of 0.3 ml min^−1^. The mobile phase consisted of (A) 10 mM ammonium acetate, pH 5 (adjusted with acetic acid), and (B) 10 mM ammonium acetate, pH 5, in 90% acetonitrile. IMP quantification was performed by negative electrospray ionisation mass spectrometry using the following parameters: capillary voltage, 2 kV; cone voltage, 20 V; source temperature, 120 °C; desolvation temperature, 400 °C; desolvation gas flow, 400 L h^−1^; and cone gas flow, 30 L h^−1^.

Intracellular IMP concentrations were calculated according to Equation (1):

(1) $${\left[\text{IMP}\right]}_{\text{in}}={\left[\text{IMP}\right]}_{\text{ex}}\frac{V_{\text{ex}}\;}{\text{OD}\cdot V_{c}\cdot \text{NC}\cdot V_{\text{cell}}}$$

Where [*IMP*]*_ex_* is the IMP concentration in the extract (μM), V*_ex_* is the extract volume (µL), OD is the optical density of the cell suspension and *V_C_* is the volume of the filtered cell suspension (mL). *N_C_* (2.3 × 10^7^ mL^−1^) represents the number of cells in 1 ml of culture at O.D0.600 = 0.1, as determined by a colony-forming unit assay. *V_cell_* (5.2 × 10^–6 ^μL) corresponds to the average volume of a rod-shaped *M. smegmatis* cell with dimensions of 5.5 × 1.1 μm.

### Enzyme kinetics

Enzyme assays were performed at the indicated pH in 50 mM Bis–Tris propane·HCl buffer containing 200 mM KCl and 2 mM DTT. Reactions were carried out at 37 ± 0.1 °C in 1 cm quartz cuvettes, and absorbance at 340 nm was continuously monitored using a Specord 200 PLUS spectrophotometer (Analytik Jena GmbH, Jena, Germany). Enzyme concentrations in the assays ranged from 5 to 20 nM.

For pH-dependent IMP and NAD^+^ kinetics, enzyme stability across the tested pH range (7.0–9.0) was verified by pre-incubating each enzyme at 500 nM in the corresponding buffer for 30 min at 30 °C, followed by activity measurement at pH 8.5 in the presence of 1 mM IMP, 3 mM NAD^+^, and 5 nM enzyme. All *Mtb*IMPDH, *Msm*IMPDH, and ΔCBS *Msm*IMPDH samples retained full activity across the tested pH interval (7.0–9.0).

Initial reaction velocities were calculated as described previously^11^. Briefly, IMPDH activity was monitored by measuring the increase in NADH absorbance at 340 nm over 10 min with 20 s intervals. The slope of the linear portion of the reaction progress curve was determined by least-squares linear regression. The initial velocity (in s^−1^) for a 1 cm pathlength cuvette was calculated according to Equation (2).

(2) $$v_{0}=\frac{\text{slope }}{[E]\cdot \varepsilon }$$

In Equation (2), the slope represents the rate of absorbance increase at 340 nm (A.s^−1^), [E] is the enzyme concentration (nM), and ε is the molar absorption coefficient of NADH at 340 nm (6.22 × 10^–6^ nM^−1^ cm^−1^).

Nonlinear regression analyses were performed in GraphPad Prism 10 (GraphPad Software Inc., San Diego, CA, USA) using the Hill equation (Equation (3) for IMP kinetics (with or without inhibitor) or the substrate inhibition model for NAD^+^ kinetics.

(3) $$v_{0}=\frac{V_{\text{lim}}\cdot {\left[\text{IMP}\right]}^{n_{H}}}{K_{0.5}^{n_{H}}+{\left[\text{IMP}\right]}^{n_{H}}}$$

Where *n_H_* is Hill coefficient, *K_0.5_* is half-constant, *V_lim_* is limiting reaction rate.

## Results and discussion

### Mycobacterial IMPDH shows highest cooperativity at physiologic pH

**Figure 1.**
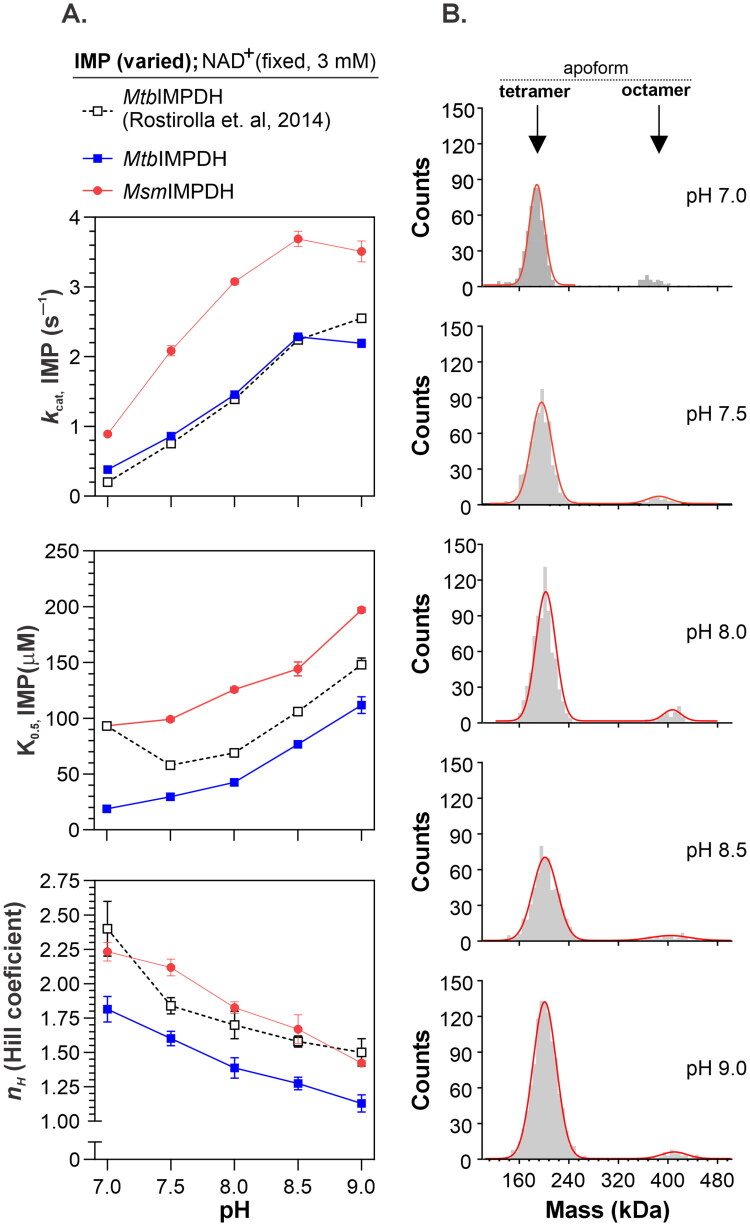
pH dependence of mycobacterial IMPDH kinetics and oligomerisation. (A) IMP kinetic parameters for *Mtb*IMPDH and *Msm*IMPDH as a function of pH. (B) Mass photometry analysis of *Msm*IMPDH oligomeric states at different pH values.

To clarify the contradictory reports on mycobacterial IMPDH cooperativity, we analysed the pH dependence of IMP kinetics within a pH range of 7.0–9.0 for both *Mtb*IMPDH and *Msm*IMPDH, and then compared their kinetic parameters (Figure 1A). Both enzymes exhibited clear cooperative IMP kinetics, displaying a similar trend in response to pH. Notably, kinetic parameters were consistent with those reported by Rostirolla et al.^17^, even though *K*_0.5_, _IMP_ and *n*_H_ values for *Mtb*IMPDH were slightly lower. However, the overall pH-dependent pattern remained comparable. While the increase in the *k*_cat, IMP_ parameter with pH can be explained by activation of the catalytic cysteine residue via proton dissociation (pK 8.2–8.3), the reason for the increase in the *K*_0.5, IMP_ value is less apparent. The simultaneous decrease in the Hill coefficient (*n*_H_) indicates that pH significantly affected IMP cooperativity. As previously reported, bacterial IMPDH activity can be influenced by its oligomeric state^12^^,^^14^. However, mass photometry analysis revealed that the pH-dependent changes in cooperativity were not accompanied by alterations in oligomeric state, as *Msm*IMPDH remained predominantly tetrameric across all tested pH conditions (Figure 1B).

**Figure 2.**
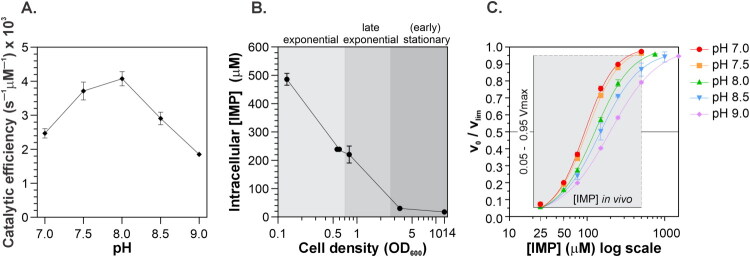
(A) Catalytic efficiency of *Msm*IMPDH, calculated from *k*_cat_ and *K*_M_ for NAD^+^ kinetics, plotted as a function of pH. (B) Intracellular IMP concentration in *M. smegmatis* at different growth stages. (C) Relative velocity (*v*_0_/*V*_max_) versus IMP concentration at different pH values. The shaded area indicates the in vivo IMP concentration range (*x*-axis) and the 0.05–0.95 *V*_max_ interval (*y*-axis).

Note that the physical interpretation of the *K*_0.5, IMP_ parameter does not allow for a direct calculation of the enzyme’s catalytic efficiency across different pH values. Therefore, we determined the pH-dependent kinetic profiles for NAD^+^. Based on the corresponding *K*_M, __NAD_^+^ and *k*_cat, __NAD_^+^ values, we then calculated and plotted the catalytic efficiency as a function of pH (Figure 2A). These data show that *Msm*IMPDH exhibited maximal catalytic efficiency within a pH range of 7.5–8.0. Interestingly, this optimal range is comparable to that reported for human IMPDH isoform 2^22^.

Placing the pH-dependent behaviour of *Msm*IMPDH into a biological context, MS-based metabolomics show that *M. smegmatis* maintained intracellular IMP concentrations between 20 and 500 μM, depending on the growth phase (Figure 2B). The intracellular pH of mycobacteria has been reported to range between 6.5 and 7.4 depending on extracellular conditions^23–25^. Notably, at pH values below 7.5, in vivo IMP concentrations falls within the range over which *Msm*IMPDH operates between 5% and 95% of its maximal velocity (Figure 2C). This range, defined by the K_0.5, IMP_ and *n*_H_ parameters, reflects biologically meaningful conditions that allow the enzyme to respond to changing substrate concentrations.

With the above data in mind, we chose a pH value of 7.4 for further *Msm*IMPDH characterisation. At this pH, the enzyme exhibits a high degree of IMP cooperativity within the range of intracellular IMP concentrations (20–500 µM) in addition to sufficient catalytic efficiency. These findings highlight the importance of selecting a pH for biochemical characterisation that reflects biologically relevant conditions.

### Mycobacterial IMPDH reaction kinetics is linked to cooperativity of catalytic domains

**Figure 3.**
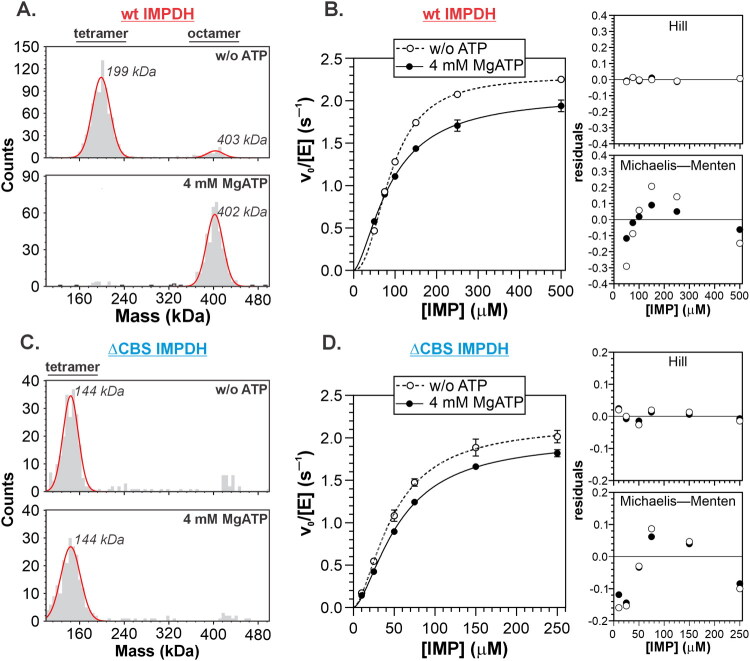
Effect of ATP and CBS domain deletion on *Msm*IMPDH. (A and C) Mass photometry analysis showing oligomeric states of full-length and ΔCBS *Msm*IMPDH with and without ATP. (B and D) IMP kinetics for full-length and ΔCBS *Msm*IMPDH with or without ATP, fitted with the Hill equation. Left panels show residuals from nonlinear regression using either the Hill or Michaelis–Menten equation.

We previously demonstrated that, unlike Class I IMPDHs, *Msm*IMPDH displays cooperative IMP kinetics as a tetramer, and exhibits only partial sensitivity to ATP binding at the CBS domain upon octamerization^11^. To assess the contribution of the CBS domain to IMP cooperativity, we examined a ΔCBS deletion mutant of *Msm*IMPDH and compared it to the WT enzyme. The effect of 4 mM ATP was confirmed, as it induced *Msm*IMPDH octamerization (Figure 3A), while the cooperative character of the initial velocity (*v*_0_) versus IMP concentration plots was only partially reduced (Figure 3B); the Hill coefficient decreased from 2.27 ± 0.04 to 1.57 ± 0.09 in the presence of ATP (Table 2). The ΔCBS mutant remained tetrameric regardless of ATP addition (Figure 3C) but still displayed a cooperative IMP kinetic profile (Figure 3D), with an *n*_H_ value of 1.64 ± 0.11 that was independent of ATP (Table 2). Notably, the K_0.5, IMP_ value of the ΔCBS variant was approximately twofold lower than that of the full-length enzyme (Table 2), a finding consistent with previous observations for the *Mtb*IMPDH counterpart^8^. The sigmoidal shape of the *v*_0_ versus [IMP] plot indicates a gradual increase in the enzyme’s affinity for IMP with increasing substrate concentration, a hallmark of cooperative behaviour. Taken together, these findings indicate that IMP cooperativity in mycobacterial IMPDHs arises from two mechanistically distinct contributions: one mediated by interactions involving CBS domains, as observed in Class I IMPDHs, and the other originating at the level of the catalytic domains within the tetramer.

**Table 2.** IMP kinetic parameters at pH 7.4.

Enzyme	Fixed NAD^+^ (mM)	Addition	*k*_cat,_ _IMP_ (s^–1^)	*K*_0.5,_ _IMP_ (µM)	*n*_H_
FL	3		2.29 ± 0.01	90 ± 1	2.27 ± 0.04
FL	3	4 mM ATP	2.06 ± 0.05	90 ± 3	1.57 ± 0.09
ΔCBS	3		2.17 ± 0.06	49 ± 2	1.64 ± 0.11
ΔCBS	3	4 mM ATP	1.99 ± 0.03	55 ± 2	1.61 ± 0.06
FL	2		2.41 ± 0.02	113 ± 2	2.22 ± 0.07
FL	2	5 µM RMP	2.07 ± 0.03	163 ± 5	1.15 ± 0.03
ΔCBS	2		2.19 ± 0.03	65 ± 2	1.69 ± 0.08
ΔCBS	2	5 µM RMP	2.02 ± 0.03	128 ± 5	1.22 ± 0.03

**Figure 4.**
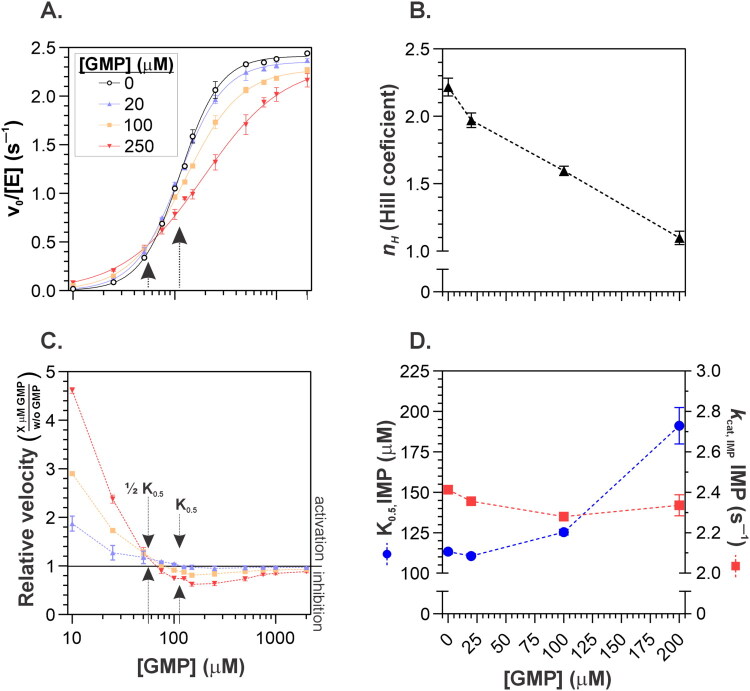
Effect of GMP on MsmIMPDH IMP kinetics. (A) *Upper panel*: Plot of initial velocity (*v*_0_) versus IMP concentration at varying GMP concentrations. *Lower panel*: Plot of relative velocities versus IMP concentration at different GMP levels, calculated as the ratio of *v*_0_ in the presence of GMP to *v*_0_ in its absence at each IMP concentration (x-axis shown on a logarithmic scale). (B) Kinetic parameters as a function of GMP concentration. *Upper panel*: Hill coefficient versus [GMP]. *Lower panel*: K_0.5, IMP_ (blue) and *k*_cat, IMP_ (red) plotted as a function of [GMP].

The cooperativity observed in the tetrameric ΔCBS *Msm*IMPDH can be explained by a model in which IMP binding to the active site of one protomer induces conformational changes in neighbouring protomers within the tetramer, thereby increasing their affinity for IMP. To investigate this hypothesis, we utilised GMP, a natural competitive inhibitor that binds to the same site as IMP within the active site^9^^,^^26^^,^^27^. Remarkably, GMP shifted the cooperative IMP kinetics of *Msm*IMPDH to a Michaelis–Menten profile (Figure 4A, lower panel). The Hill coefficient decreased in a manner inversely proportional to GMP concentration, dropping from 2.21 ± 0.07 to 1.10 ± 0.05 at 250 µM GMP (Figure 4B, upper panel). As expected for a competitive inhibitor, GMP did not alter the *k*_cat_ value but increased K_0.5,IMP_, consistent with its mode of inhibition (Figure 4B, bottom panel). The decrease in *n*_H_ can be attributed to the asymmetric effect of GMP at different IMP concentrations (Figure 4A, lower panel): at low IMP concentrations (below half the K_0.5, IMP_ of the uninhibited reaction), GMP increased the reaction velocity in a concentration-dependent manner, whereas at high IMP concentrations (above K_0.5, IMP_), the opposite effect was observed.

This effect of GMP aligns with our proposed model of IMP cooperativity mediated by interactions between the catalytic subunits of *Msm*IMPDH. Counterintuitively, GMP enhances the reaction rate at low IMP concentrations, reflecting its ability to increase apparent IMP affinity in neighbouring subunits. Conversely, GMP inhibits the enzyme more effectively at higher IMP concentrations, consistent with its role as an IMP analogue. When the enzyme is partially saturated with IMP (at around 50%), the remaining unoccupied subunits exhibit a higher affinity for GMP. As a result, GMP binds more efficiently and inhibits *Msm*IMPDH to a greater extent under conditions of intermediate IMP saturation, compared to conditions where IMP levels are low.

While the reaction mechanism of IMPDH, which involves extensive rearrangements of flexible loops near the active site, is well-established^9^^,^^28^, the mechanistic basis of its cooperative behaviour is less conclusive. We recently proposed that one mechanism may involve the C-terminal regions of *Msm*IMPDH, which interact with the active sites of adjacent subunits within the tetramer^29^. These interactions are essential for coordinating a K^+^ ion that stabilises the proper conformation of the active site. Folding of the C-termini is directly linked to substrate IMP binding, providing a plausible mechanism that partially accounts for the observed cooperativity of *Msm*IMPDH at the level of the catalytic domains within the tetramers.

### IMP-based synthetic inhibitors may result in partial activation of IMPDH

**Figure 5.**
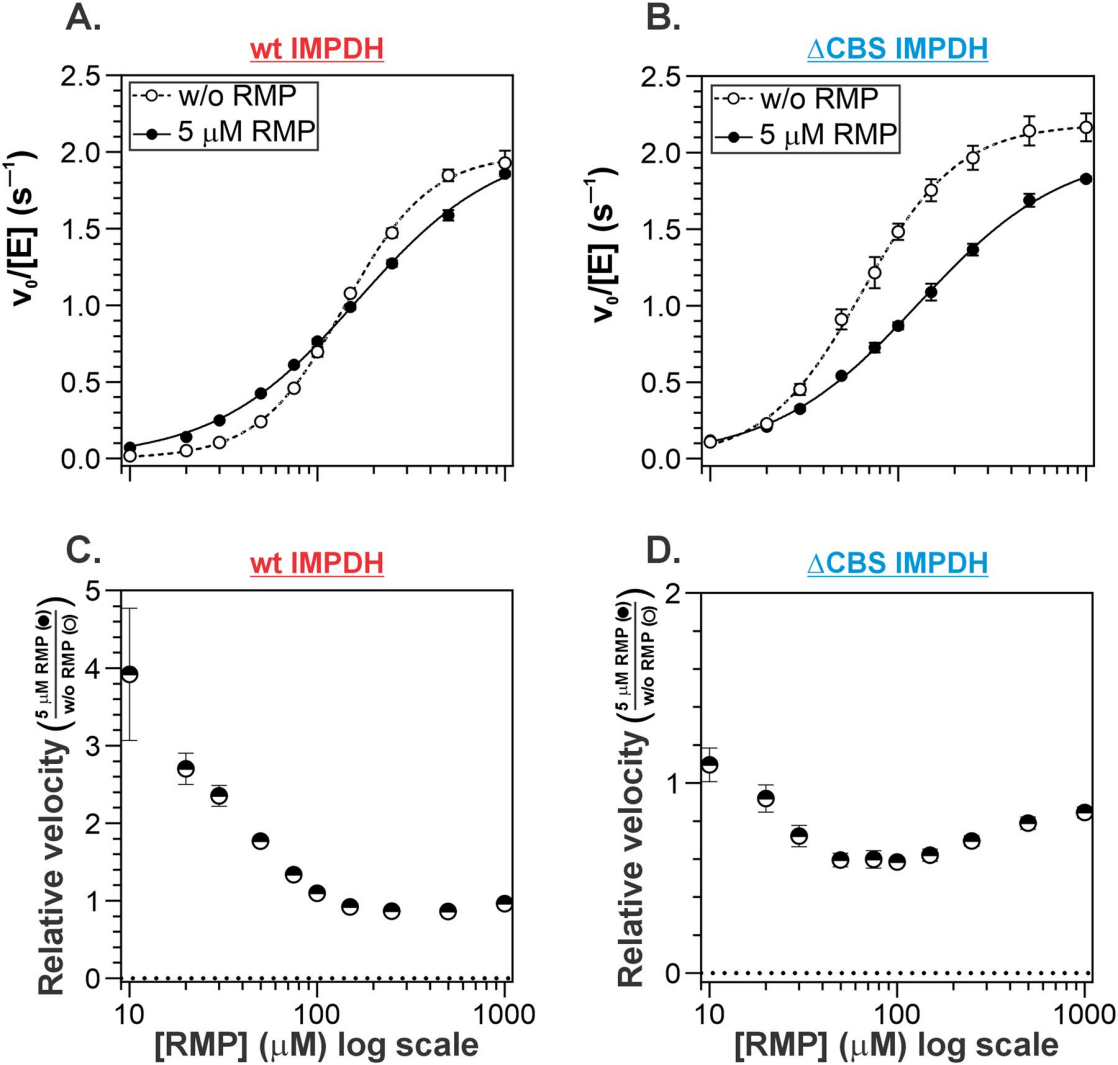
Effect of RMP on full-length and ΔCBS *Msm*IMPDH. (A and B) Initial velocity (*v*_0_) versus IMP concentration for (A) full-length and (B) ΔCBS *Msm*IMPDH. Filled and empty circles indicate reactions with and without 5 μM RMP, respectively. (C and D) Relative velocities versus IMP concentration for (C) full-length and (D) ΔCBS *Msm*IMPDH, calculated as the ratio of *v*_0_ in the presence of RMP to *v*_0_ in the absence of RMP.

The effect of GMP on the allosteric IMP kinetics of mycobacterial IMPDH suggests that IMP-competitive inhibitors may cause unintended activation or reduced inhibition of the enzyme under certain conditions. To test this hypothesis, we examined the effect of 5 µM ribavirin 5′-monophosphate (RMP), a well-characterised IMP analogue and established IMPDH inhibitor^30^ (Figure 5(A,B)). Our data confirm that RMP exerted a mechanistic effect similar to that of GMP. Specifically, RMP decreased the Hill coefficient (*n*_H_) to 1.15 ± 0.03 for the full-length enzyme and to 1.22 ± 0.03 for the ΔCBS variant (Table 2). As observed with GMP, the reduction in cooperativity was due to the disproportionate effect of RMP at low versus high IMP concentrations in both WT and ΔCBS *Msm*IMPDH variants (Figure 5(C,D)).

**Figure 6.**
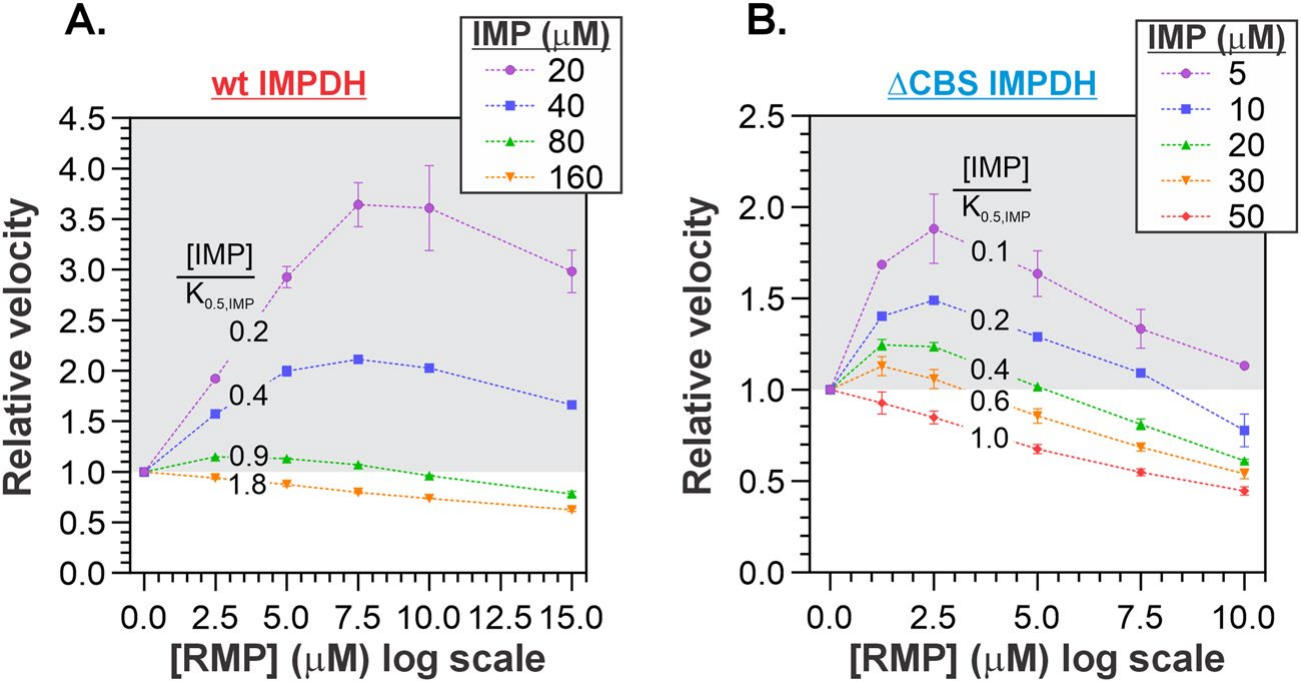
Effect of RMP on *Msm*IMPDH at fixed IMP concentrations. Relative velocity versus RMP concentration for (A) WT and (B) ΔCBS *Msm*IMPDH at varying IMP concentrations.

We next examined the effect of RMP on both WT and ΔCBS *Msm*IMPDH in more detail using a reverse experimental approach designed to minimise variability caused by differences in substrate or enzyme concentrations inherent to traditional *v*_0_ versus IMP assays. In this setup, varying concentrations of RMP were added to reaction mixtures containing fixed concentrations of IMP, NAD^+^, and the enzyme; initial reaction rates were measured in parallel (Figure 6). These measurements confirmed the activating effect of RMP at IMP concentrations below half the K_0.5, IMP_ value, as well as a clearly inhibitory effect at concentrations above K_0.5, IMP_, consistent with the expected competitive inhibition mechanism. Collectively, these results demonstrate that accurate assessment of IMP-competitive inhibitors targeting mycobacterial IMPDH requires careful selection of substrate concentrations that reflect physiologically relevant in vivo conditions. This is essential in order to avoid any misinterpretation of inhibitor effects due to the enzyme’s cooperative kinetics.

**Figure 7.**
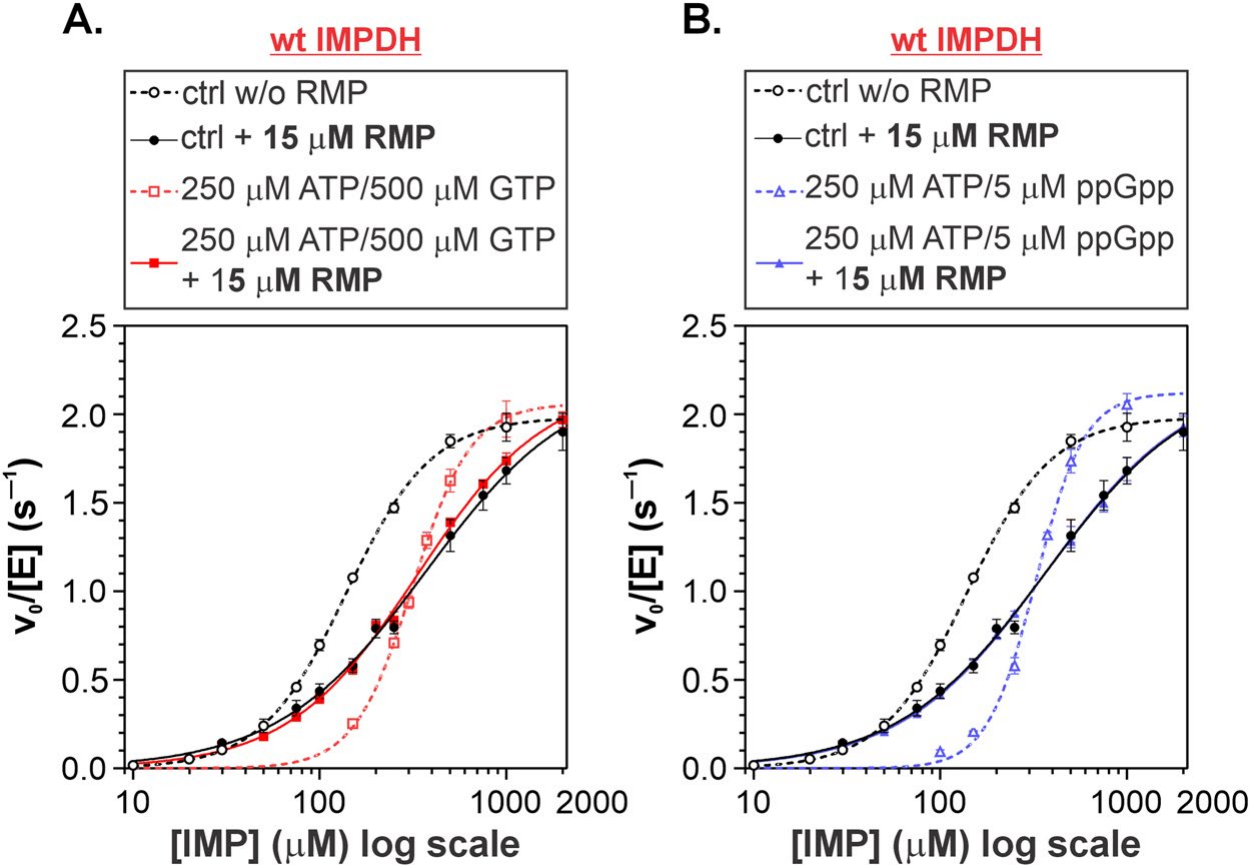
Impact of 15 µM RMP on (A) GTP- and (B) ppGpp-mediated inhibition of *Msm*IMPDH.

Mycobacterial IMPDH is regulated by the natural allosteric inhibitors GTP and ppGpp, which bind to the CBS domain^11^. We next investigated how this allosteric inhibition is influenced by the presence of an IMP-competitive inhibitor. To this end, we measured the kinetic profile of *Msm*IMPDH in the presence of 5 μM ppGpp or 500 μM GTP, with or without 15 μM RMP (Figure 7A,B). Surprisingly, under these conditions, RMP effectively neutralised the inhibitory effects of both guanine nucleotide allosteric inhibitors. This finding can be explained by RMP mimicking the effect of IMP binding, which has been shown to extend the compressed, inactive *Msm*IMPDH octamer^11^ and relieve inhibition. This interplay between IMP-competitive and allosteric inhibitors introduces an additional layer of complexity that should be considered when evaluating the effects of IMPDH inhibitors.

## Limitations of the study

The objective of this study was to elucidate the cooperative nature of mycobacterial IMPDH kinetics and its relationship to the CBS domain, as well as to natural allosteric and synthetic competitive inhibitors in vitro. The experiments were conducted using recombinant IMPDH heterologously expressed in *E. coli*, as is standard in biochemical and drug discovery studies. However, it is important to acknowledge that under cellular conditions, enzymes may undergo post-translational modifications that influence their quaternary structure and catalytic properties including cooperativity. Moreover, the presence of as-yet-unidentified allosteric regulators in the native cellular environment may further modulate IMPDH activity, either positively or negatively. In light of these considerations, the results of the in vitro inhibition assays should be interpreted with caution and ultimately validated through in vivo experiments.

## Conclusions

In summary, our study reveals that the cooperative behaviour of mycobacterial IMPDH adds substantial nuance to kinetic experiments. We demonstrate that *Msm*IMPDH cooperativity arises from two mechanistically distinct components: one mediated by the CBS domain, and the other driven by interactions within the catalytic domains of the tetramer. This latter mechanism is directly linked to IMP binding, which enhances substrate affinity at neighbouring active sites. Importantly, this mode of cooperativity can produce paradoxical activation effects by IMP-competitive inhibitors under certain conditions, highlighting the need for careful experimental design and interpretation.

Based on the above findings, we propose the following considerations for the design and interpretation of kinetic experiments aimed at evaluating IMPDH inhibitors:

- pH conditions should be carefully selected to support both the cooperative behaviour and sufficient catalytic efficiency of the enzyme. We recommend using physiologically relevant pH values in the range of 6.5–7.4, reflecting the intracellular environment of mycobacteria.
- The full-length WT enzyme should be used to account for cooperative contributions from both the CBS domain and the catalytic core.
- The concentration of the substrate IMP should be chosen with care, particularly to avoid misleading activation effects that may arise when competitive inhibitors are tested at substrate concentrations below 50% saturation.
- The effects of competitive inhibitors should be interpreted in the context of natural allosteric regulators, considering possible interactions with effectors such as GTP and ppGpp.

## Data Availability

The source data for all biochemical assays have been deposited in the Zenodo repository and are available at the following URL: https://doi.org/10.5281/zenodo.16992680
